# T2-Weighted Image-Based Radiomics Signature for Discriminating Between Seminomas and Nonseminoma

**DOI:** 10.3389/fonc.2019.01330

**Published:** 2019-11-28

**Authors:** Peipei Zhang, Zhaoyan Feng, Wei Cai, Huijuan You, Chanyuan Fan, Wenzhi Lv, Xiangde Min, Liang Wang

**Affiliations:** ^1^Department of Radiology, Tongji Hospital, Tongji Medical College, Huazhong University of Science and Technology, Wuhan, China; ^2^Julei Technology, Wuhan, China

**Keywords:** magnetic resonance imaging, T2-weighted imaging, testicular neoplasms, testicular germ cell tumors, radiomics

## Abstract

**Objective:** To evaluate the performance of a T2-weighted image (T2WI)-based radiomics signature for differentiating between seminomas and nonseminomas.

**Materials and Methods:** In this retrospective study, 39 patients with testicular germ-cell tumors (TGCTs) confirmed by radical orchiectomy were enrolled, including 19 cases of seminomas and 20 cases of nonseminomas. All patients underwent 3T magnetic resonance imaging (MRI) before radical orchiectomy. Eight hundred fifty-one radiomics features were extracted from the T2WI of each patient. Intra- and interclass correlation coefficients were used to select the features with excellent stability and repeatability. Then, we used the minimum-redundancy maximum-relevance (mRMR) and the least absolute shrinkage and selection operator (LASSO) algorithms for feature selection and radiomics signature development. Receiver operating characteristic curve analysis was used to evaluate the diagnostic performance of the radiomics signature.

**Results:** Five features were selected to build the radiomics signature. The radiomics signature was significantly different between the seminomas and nonseminomas (*p* < 0.01). The area under the curve (AUC), sensitivity, and specificity of the radiomics signature for discriminating between seminomas and nonseminomas were 0.979 (95% CI: 0.873–1.000), 90.00 (95% CI: 68.3–98.8), and 100.00 (95% CI: 82.4–100.0), respectively.

**Conclusion:** The T2WI-based radiomics signature has the potential to non-invasively discriminate between seminomas and nonseminomas.

## Introduction

Testicular cancer represents 1% of neoplasms and 5% of urological tumors in males. However, testicular cancer is the most common malignancy among men aged between 14 and 44 years (1, 2). Statistics show that there were 71,105 new cases and 9,507 deaths of testicular cancer worldwide in 2018 (3). Approximately 90–95% of testicular cancers are testicular germ cell tumors (TGCTs), which are split into two broad categories: seminomas and nonseminomas (4).

Radical orchiectomy is the main treatment for testicular tumors and can be supplemented by radiotherapy and chemotherapy (4, 5). In view of the different sensitivities of seminomas and nonseminomas to radiotherapy and chemotherapy, characterizing the histologic type of testicular tumors is of great importance (6–8). For patients undergoing orchidectomy, the differentiation of seminomas from nonseminomas would not affect patient management. However, the information gained preoperatively might help physicians to explain the patient's condition and tumor prognosis before surgery, which would help decrease the patient's anxiety. However, for patients who are unwilling to undergo orchiectomy, the seminomas, and nonseminomas must be identified by other non-invasive means, such as imaging examinations, because the guidelines do not recommend that patients with suspected testicular tumors undergo punctures in order to avoid tumor spread and metastasis (5). Therefore, several studies have evaluated the value of sonography or magnetic resonance imaging (MRI) for the non-invasive differentiation of seminomas from nonseminomas (4, 9–11).

Currently, ultrasonography (US) is the initial imaging method for confirming the existence of a testicular mass (5, 12). MRI has emerged as a valuable modality that can be an alternative diagnostic tool, especially in cases of non-diagnostic or equivocal sonographic findings (13). Compared to US, MRI can provide more abundant anatomical and functional information and is less dependent on operator technique. Some MRI features of TGCTs have been found to closely correlate with histopathologic characteristics (4, 9). T2-weighted imaging (T2WI) is an essential component of MRI in oncology. Some previous studies reported that seminomas and nonseminomas have different features on T2WI (8, 9). Most of the previous studies only used qualitative features or limited quantitative features, which may not fully explore the potential value of MRI.

Radiomics uses advanced image processing techniques to extract a large number of quantitative features from imaging data (14–16). It has been applied to various diseases such as lung and head-and-neck cancer (17), gastric cancer (18), colorectal cancer (19), liver fibrosis (20), and prostate cancer (21), etc., and remarkably encouraging results have been reported. However, to date, no study has applied radiomics to the evaluation of testicular diseases.

The purpose of our study was to investigate whether a T2WI-based radiomics signature could differentiate seminomas from nonseminomas.

## Materials and Methods

### Patient Information

**Figure 1:**
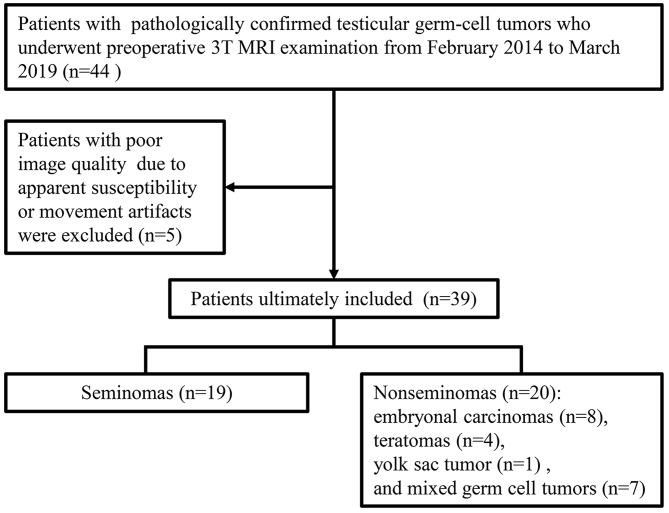
Flow chart of patients' inclusion and exclusion.

Our institutional review board approved this retrospective study. From February 2014 to March 2019, patients were included according to the following inclusion criteria (Figure 1): (a) had scrotal lesions on sonography or physical examination, (b) underwent a preoperative 3T MRI examination, (c) underwent radical orchiectomy, and (d) had pathologically confirmed TGCTs. Patients were excluded if apparent susceptibility or movement artifacts existed on the MR images. A total of 39 men (age range, 18–61 years; median age, 29 years) with 39 lesions were included. Nineteen tumors were pathologically confirmed as seminomas, and 20 tumors were pathologically confirmed as nonseminomas. The patients with nonseminomas had embryonal carcinomas (*n* = 8), teratomas (*n* = 4), yolk sac tumor (*n* = 1), and mixed germ cell tumors (*n* = 7) [embryonal carcinomas and teratomas (*n* = 3), seminoma and embryonal carcinoma (*n* = 1), teratoma, yolk sac tumor and embryonal carcinoma (*n* = 1), seminoma, teratoma and yolk sac tumor (*n* = 1), and seminoma, embryonal carcinoma, teratoma, and yolk sac tumor (*n* = 1)]. The classification of the tumor types in the current study was based on the NCCN guideline (22).

### MRI Protocol

All the MR images were acquired with a 3T MR scanner (MAGNETOM Skyra, Siemens Healthcare, Erlangen, Germany) and an 18-element body matrix coil in combination with a 32-channel spine coil. The patients were positioned in a feet-first supine position. Transverse, sagittal and coronal T2-weighted turbo spin-echo sequences with the following parameters were used: repetition time/echo time (TR/TE) range 6500-6870/104 ms, slice thickness of 3~5 mm, interslice gap of 0~0.5 mm, field of view (FOV) of 180 × 180 mm^2^, and a matrix of 384 × 320. Transverse T1-weighted turbo spin-echo sequences were acquired with the following parameters: TR of 750 ms, TE of 13 ms, slice thickness of 3~5 mm, interslice gap of 0~0.5 mm, FOV of 300×300 mm^2^, and matrix of 320 × 240. Diffusion-weighted imaging (DWI) and dynamic contrast enhanced (DCE) sequences were performed for some patients, but these images were not included in the analysis due to the limited number of scans.

### MRI Segmentation and Radiomics Feature Extraction

ITK-SNAP software (version 3.4.0; www.itksnap.org) was used for manual segmentation. Preoperative transverse T2WI was obtained for image analysis. A three-dimensional volume of interest (VOI) covering the tumor was delineated by stacking regions of interest slice-by-slice on the transverse T2WI. Manual segmentation of the tumors on the images was initially performed by a radiologist (Reader 1). Twenty patients were randomly selected from the study cohort. One month later, Reader 1 performed a second segmentation of the 20 patients to assess the intraobserver reproducibility. Another radiologist (Reader 2) performed a manual segmentation of these patients independently to assess the interobserver reproducibility. Both readers were blinded to the histologic results.

The radiomics features were extracted using the PyRadiomics library (https://github.com/Radiomics/pyradiomics.git, version 2.1.2) in Python (version 3.7.0). PyRadiomics is a flexible open-source platform capable of extracting a large panel of engineered features from medical images (23). For the feature extraction method, please reference the PyRadiomics documentation (https://pyradiomics.readthedocs.io/en/latest/). All MRI data were subjected to images normalization and resampled to the same resolution (0.46875 × 0.46875 × 3 mm) before feature extraction. A total of 851 radiomics features were extracted, including the following four groups: 14 shape features, 18 first-order intensity statistics features, 75 texture features [Gray Level Co-occurrence Matrix (24), Gray Level Size Zone Matrix (16), Gray Level Run Length Matrix (16), Neighboring Gray Tone Difference Matrix (5), and Gray Level Dependence Matrix (14)], and 744 wavelet features.

### Statistical Analysis

As high-dimensional features were extracted in the current study, we performed a feature dimension reduction process to select the most relevant features for the classification of testicular lesions to construct a radiomics signature. Features selection included the following steps. First, we used the intra- and interclass correlation coefficient (ICC) to assess the effects of the manual segmentation variations on the value of the features. The ICC was calculated for each radiomics feature. Features with good agreement (ICC ≥ 0.8) were regarded as robust features and selected for the following analyses. Second, we compared all the features between seminomas and nonseminomas using the Mann-Whitney *U* test for non-normally distributed features or the independent *t*-test for normally distributed features. Features with *p* < 0.05 were considered significant variables and selected. To control the false-positive rate in multiple comparisons, the false discovery rate-adjusted *p*-value was used in the Mann-Whitney *U* test and the independent *t*-test (24). Third, spearman's correlation coefficient was used to compute the relevance and redundancy of the features. Redundant features indicated by a Spearman's correlation coefficient ≥ 0.8 were eliminated. Fourth, we applied the maximum relevance minimum redundancy (mRMR) algorithm to assess the relevance and redundancy of the remaining features (25). The mRMR algorithm was used to select the most relevant features for the classification of testicular lesions, avoiding redundancy between features. By using the mRMR method, the features were ranked according to their relevance-redundancy scores (mRMR scores). The mRMR score of a feature is defined as the mutual information between the status of the lesions and this feature minus the average mutual information of previously selected features and this feature. The top 10 features with high-relevance and low-redundancy were selected for the following analyses. Fifth, the 10 features selected by the above steps were applied to least absolute shrinkage and selection operator (LASSO) logistic regression model (26). The LASSO logistic regression model with 5-fold cross-validation was adopted for further features selection and radiomics signature construction. LASSO is a regression analysis method that performs feature selection and regularization to improve the mode prediction accuracy and interpretability. Some candidate features coefficients were shrunk to zero and the remaining variables with non-zero coefficients were selected by LASSO. Then, the selected features were linearly combined to construct a radiomics signature.

**Figure 2:**
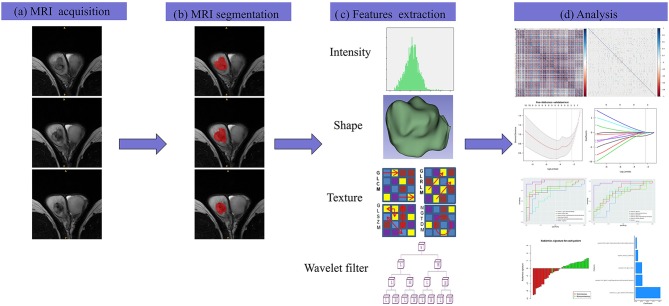
The framework for the radiomics workflow. **(a)** All patients were scanned with preoperative MRI. **(b)** Tumors were delineated by stacking regions of interest slice-by-slice on the transverse T2WI. **(c)** Radiomics features were extracted from the T2WI in a high-throughput manner. **(d)** Data analysis for the features selection and a radiomics signature construction.

The differences in the radiomics signature between seminomas and nonseminomas were compared using the Mann-Whitney *U* test. The diagnostic performance of the radiomics signature was evaluated using the receiver operating characteristic (ROC) curve. The area under the curve (AUC), sensitivity, and specificity were calculated. In addition, the diagnostic performance of the top 10 features selected from mRMR was also evaluated using ROC curve analysis. An overview of the radiomics signature development process is presented in Figure 2.

The statistical analyses were performed using R software (version 3.3.4; https://www.r-project.org). The following R packages were used: the “corrplot” package was used to calculate Spearman's correlation coefficient; the “mRMRe” package was used to implement the mRMR algorithm; the “glmnet” was used to perform the LASSO logistic regression model, and the “pROC” package was used to construct the ROC curve.

## Results

**Figure 3:**
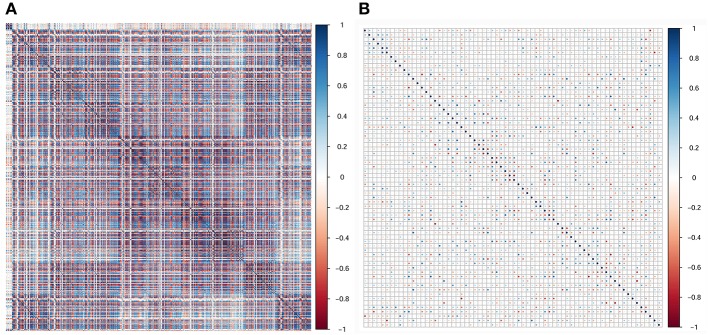
Correlation matrix heatmaps of the features before **(A)** and after **(B)** correlation filtering. Before correlation filtering, a mass of redundant features with high correlation coefficients existed.

**Figure 4:**
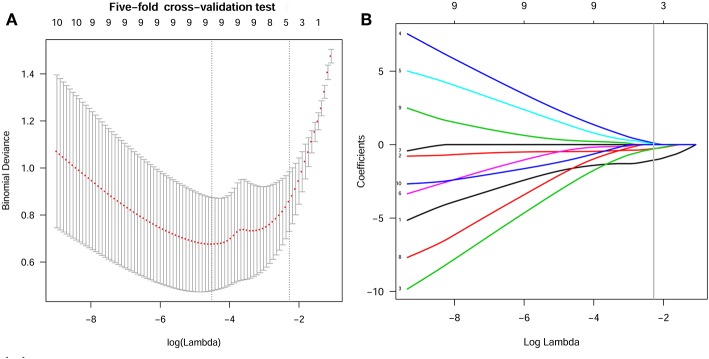
Features selection using the LASSO algorithm. **(A)** Selection of the tuning parameter (Lambda) in the LASSO model using 5-fold cross-validation. Binomial deviances from the LASSO regression cross-validation model were plotted as a function of log(Lambda). The dotted vertical line at the right was drawn at the optimal value based on the minimum criteria and the 1-standard error rule (the 1-SE criteria). An optimal Lambda value of 0.102 with log(Lambda) = −2.280 and 5 non-zero coefficients were selected. **(B)** LASSO coefficient profiles of the 10 texture features. A vertical line was drawn at the optimal value selected using the 5-fold cross-validation process in **(A)**. The 5 features with non-zero coefficients were included to construct the radiomics signature.

**Figure 5:**
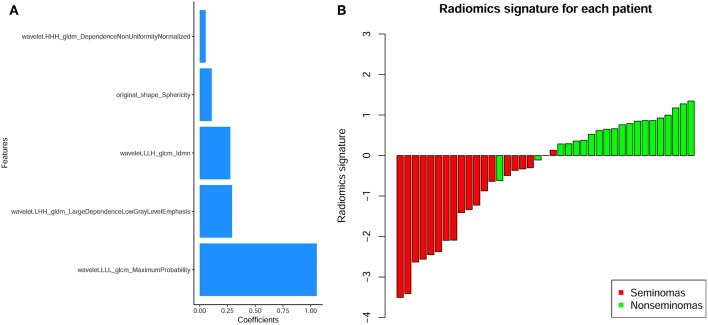
**(A)** The contribution of the features to the radiomics signature. The histogram shows the contribution of the five features with non-zero coefficients to the radiomics signature. The features that contributed to the radiomics signature are plotted on the y-axis, and their coefficients in the LASSO Cox analysis are plotted on the x-axis. **(B)** Bar charts of the radiomics signature for each patient. The red bars indicate the radiomics signature of seminomas, while the light green bars indicate the radiomics signature of non-seminomas.

In the current study, 851 radiomics features were extracted from the T2WI of each patient. Seven hundred eighty features with an ICC ≥ 0.8 were further selected. Two hundred twenty-seven non-significant features were first eliminated using univariate analysis. After removing the redundant features using a Spearman's correlation coefficient threshold value of 0.8, a total of 67 features with low correlation remained. The correlation matrix heatmaps of the features before and after correlation filtering are shown in Figure 3. The features were ranked according to their mRMR scores. The top 10 features were selected using the mRMR algorithm (Table 1). Through the 5-fold cross-validation of the LASSO algorithm, five features with non-zero coefficients were included to construct the radiomics signature. The feature selection process using the LASSO algorithm is shown in Figure 4. The calculation formula to construct the radiomics signature is shown in Table 2. The contribution of the five features to the radiomics signature is shown in Figure 5A. The radiomics signature of each patient is shown in Figure 5B.

**Table 1:** The top 10 features selected by mRMR.

**Features**	**mRMR scores**	**Groups**
wavelet.LLL_glcm_MaximumProbability	0.31769474	Wavelet feature
wavelet.LLH_glcm_Idmn	0.08877716	Wavelet feature
wavelet.LHH_gldm_LargeDependenceLowGrayLevelEmphasis	0.07167124	Wavelet feature
original_shape_Sphericity	0.07024193	Shape feature
wavelet.HHH_gldm_DependenceNon-UniformityNormalized	0.07355068	Wavelet feature
wavelet.LHL_glcm_Idn	0.04066711	Wavelet feature
wavelet.LLH_gldm_DependenceEntropy	0.04644461	Wavelet feature
wavelet.LLH_glcm_MCC	0.02630265	Wavelet feature
wavelet.LHL_glrlm_LongRunHighGrayLevelEmphasis	0.02301324	Wavelet feature
wavelet.LHL_firstorder_Skewness	0.02354773	Wavelet feature

**Table 2:** Calculation formula for the radiomics signature.

**Variables**	**Coefficients**
Intercept	−0.04258474
wavelet.LLL_glcm_MaximumProbability	−1.05440198
wavelet.LLH_glcm_Idmn	−0.27559477
wavelet.LHH_gldm_LargeDependenceLowGrayLevelEmphasis	−0.29108858
original_shape_Sphericity	0.10820225
wavelet.HHH_gldm_DependenceNon-UniformityNormalized	0.05352220

**Figure 6:**
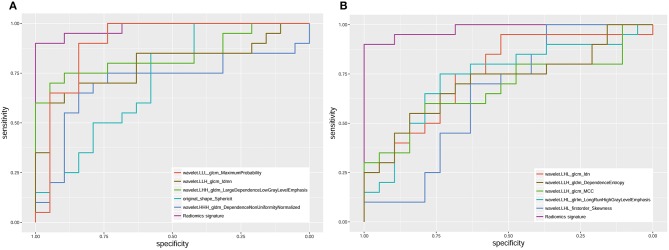
ROC analysis of the radiomics signature and 10 features [**(A)** the top 5; **(B)** the bottom five)] selected from mRMR. The AUC of the radiomics signature was 0.979 (95% CI: 0.873–1.000).

The radiomics signature was significantly different between seminomas and nonseminomas (*p* < 0.01). The ROC curves of the radiomics signature and the top 10 features selected from mRMR for discriminating between seminomas and nonseminomas are shown in Figure 6 and Table 3. The AUC, sensitivity, and specificity of the radiomics signature were 0.979 (95% CI: 0.873–1.000), 90.00 (95% CI: 68.3–98.8), and 100.00 (95% CI: 82.4–100.0), respectively. The AUC of the radiomics signature was relativity higher than the AUCs of the top 10 features selected from mRMR.

**Table 3:** ROC analysis of the features selected from mRMR.

**Features**	**AUC (95% CI)**	**Sensitivity (95% CI)**	**Specificity (95% CI)**
Radiomics signature	0.979 (0.873–1.000)	90.00 (68.3–98.8)	100.00 (82.4–100.0)
wavelet.LLL_glcm_MaximumProbability	0.903 (0.764–0.974)	90.00 (68.3–98.8)	84.21 (60.4–96.6)
wavelet.LLH_glcm_Idmn	0.792 (0.632–0.905)	60.00 (36.1–80.9)	94.74 (74.0–99.9)
wavelet.LHH_gldm_LargeDependenceLowGrayLevelEmphasis	0.839 (0.687–0.937)	70.00 (45.7–88.1)	94.74 (74.0–99.9)
original_shape_Sphericity	0.718 (0.552–0.850)	85.00 (62.1–96.8)	57.89 (33.5–79.7)
wavelet.HHH_gldm_DependenceNonUniformityNormalized	0.703 (0.535–0.838)	65.00 (40.8–84.6)	84.21 (60.4–96.6)
wavelet.LHL_glcm_Idn	0.758 (0.594–0.880)	95.00 (75.1–99.9)	52.63 (28.9–75.6)
wavelet.LLH_gldm_DependenceEntropy	0.711 (0.543–0.844)	55.00 (31.5–76.9)	84.21 (60.4–96.6)
wavelet.LLH_glcm_MCC	0.679 (0.510–0.819)	55.00 (31.5–76.9)	84.21 (60.4–96.6)
wavelet.LHL_glrlm_LongRunHighGrayLevelEmphasis	0.737 (0.571–0.865)	75.00 (50.9–91.3)	73.68 (48.8–90.9)
wavelet.LHL_firstorder_Skewness	0.647 (0.478–0.793)	100.00 (83.2–100.0)	36.84 (16.3–61.6)

## Discussion

In this study, an MRI-based radiomics signature was established to preoperatively discriminate between seminomas and nonseminomas. Our results showed that the radiomics signature could provide an excellent diagnostic performance (AUC = 0.979) by employing a large number of quantitative imaging features (851 features were extracted).

Non-invasively discriminating between seminomas and nonseminomas is of great significance. MRI has been proposed as a valuable supplemental imaging technique for characterizing testicular tumors (4, 9, 11). Tsili AC et al. enrolled 21 patients (10 seminomas and 11 nonseminomas) to investigate the value of MRI for differentiating seminomas from nonseminomas (9). Their results showed that the MRI findings led to a correct histologic diagnosis in 19 (91%) of 21 cases and the researchers concluded that tumor heterogeneity on MRI is indicative of nonseminomas. Another study including 15 seminomas and 11 nonseminomas showed that the mean apparent diffusion coefficient (ADC) values of seminomas were significantly lower than those of nonseminomas, while no significant differences were observed in DCE between seminomas and nonseminomas (4). Min et al. included 14 seminomas and 10 nonseminomas to assess the value of whole-tumor ADC histogram parameters for discriminating between seminomas and nonseminomas (11). Their results showed that the 10th percentile ADC value yielded the highest diagnostic performance. Although some positive results for distinguishing seminomas from nonseminomas have been reported, most previous studies used only some qualitative features or limited quantitative features, which may not fully explore the potential information of MRI, and no established prediction model has been built. In contrast to the above studies, in our study, a large number of quantitative radiomics features were extracted from the images and the most useful features were selected to construct a radiomics signature. Moreover, the sample size included in our study was relatively larger than that in previous studies.

Medical imaging provides valuable information for the diagnosis and evaluation of diseases. The conventional methods only use some qualitative features observable by the naked eyes or basic quantitative features, which cannot fully mine potential information from the images. Radiomics may help find potentially valuable information through the high-throughput extraction of quantitative features (14, 15). The newly proposed radiomics method has been successfully applied to various diseases (17, 18, 27–30). In a recent study, Lewin et al. applied radiomics to predict the pathology of postchemotherapy retroperitoneal nodal masses in germ cell tumors (27). Their results showed that the discriminative accuracy, sensitivity, and specificity of radiomics to identify GCT/teratoma vs. fibrosis was 72, 56.2, and 81.9%, respectively. When combined with clinical variables, the accuracy improved to 88%. In another study, Dong et al. suggested that a CT-based radiomic nomogram had excellent predictive ability for occult peritoneal metastasis in advanced gastric cancer patients (18). In our study, we used radiomics analysis to extract 851 features from T2WI and constructed a radiomics signature that includes features with excellent stability and reproducibility. Our results showed that the radiomics signature provides excellent efficiency for discriminating seminomas from nonseminomas. The AUC, sensitivity, and specificity of the radiomics signature were 0.979, 90.00, and 100.00, respectively. The AUC of the radiomics signature was 7.6–33.2% higher than the AUCs of the top 10 features selected from mRMR.

In this study, we only included T2WI for analysis, because T2WI is an essential component of testicular MRI with high contrast and spatial resolution. Previous studies have reported that seminomas and nonseminomas have different characteristics on T2WI (9). The presence of a relatively homogeneous testicular mass with low signal intensity on T2WI is considered indicative of seminomas. On the other hand, tumor heterogeneity is the most valuable finding in the characterization of nonseminomas. Although some studies have demonstrated the value of DWI and DCE in the characterization of testicular tumors (4, 11), these sequences have their limitations. The geometric distortion, susceptibility, and signal intensity dropout of DWI on tissue-air boundaries, such as the prostate, scrotum, and thyroid gland, are remarkable. Moreover, the DWI sequence usually has a low spatial resolution. These factors will limit the application and efficiency of DWI in characterizing testicular tumors. In recent years, some new techniques have been applied to DWI sequence to reduce geometric distortion and susceptibility artifacts, as well as to improve image resolution. However, few of these techniques had been used in testes; we will explore the value of new DWI techniques in testes in future studies (31–33). DCE-MRI usually requires the injection of a gadolinium-based contrast agent, which may increase the patient's risk for nephrogenic systemic fibrosis. Considering the above reasons and the limited sample size, we did not include DWI and DCE in the analysis.

There are some limitations in this study. First, our sample size was small. Although the number of patients included was higher than that of most previous studies, the sample size was still relatively small due to the low morbidity of testicular tumors. Further large-scale and multicenter studies are therefore warranted to obtain high-level evidence for clinical application. Second, rather than an independent validation cohort, internal validation was used in the current study, because there is insufficient data available to create an independent training cohort and a validation cohort. In this case, a fair way to accurately estimate the diagnostic performance of the radiomics signature is to use cross-validation (34). Third, the MRI sequences employed similar parameters but slightly varied slice numbers and thicknesses to cover some large lesions. To this end, we resampled the images before feature extraction to decrease the variability of the radiomics features extracted from the MRI sequences (35).

In conclusion, in the present study, we established a radiomics signature based on the features extracted from T2WI to characterize TGCTs. The radiomics signature provides a non-invasive and quantitative method to differentiate between seminomas from nonseminomas. Further studies are warranted to validate our initial results.

## Data Availability Statement

The raw data supporting the conclusions of this manuscript will be made available by the authors, without undue reservation, to any qualified researcher.

## Ethics Statement

The studies involving human participants were reviewed and approved by Tongji Hospital, Tongji Medical College, Huazhong University of Science and Technology institutional review board. The patients/participants provided their written informed consent to participate in this study.

## Author Contributions

LW, XM, and PZ conception and design. PZ, ZF, WC, HY, CF, and WL acquisition of data. PZ and XM data processing. PZ, ZF, and XM analysis and interpretation of data. LW and XM supervision. PZ and ZF drafting the article. LW, XM, WC, HY, CF, and WL revising article critically for important intellectual content. All authors final approval of the manuscript.

### Conflict of Interest

WL was employed by company Julei Technology. The remaining authors declare that the research was conducted in the absence of any commercial or financial relationships that could be construed as a potential conflict of interest.
